# Preliminary In Vitro Cytotoxicity, Mutagenicity and Antitumoral Activity Evaluation of Graphene Flake and Aqueous Graphene Paste

**DOI:** 10.3390/life12020242

**Published:** 2022-02-07

**Authors:** Stefania Lamponi

**Affiliations:** Department of Biotechnology, Chemistry and Pharmacy and SienabioACTIVE, University of Siena, Via Aldo Moro 2, 53100 Siena, Italy; Stefania.Lamponi@unisi.it; Tel.: +39-0577-232110; Fax: +39-0577-234254

**Keywords:** graphene, cytotoxicity, mutagenicity, antitumoral activity, cell morphology

## Abstract

This study aimed to determine the in vitro cytotoxicity and mutagenicity of graphene flake (GF) and aqueous graphene paste (AGP) in order to evaluate their potential for application as biomaterials. Furthermore, their antitumor activity against adherent and suspended cells, namely, human breast adenocarcinoma cells (MDA-MB-231), and human monocytes from histiocytic lymphoma (U-937), was investigated. The results demonstrated that GF reduced the viability and proliferation of NIH3T3 immortalized murine fibroblasts for concentrations >0.8 µg/mL and incubation times of 48 and 72 h. AGP showed no toxic effects in any of the tested concentrations and incubation times. The same results were obtained for MDA-MB-231 cells. The viability of the U-937 cells was not affected by either GF or AGP. The Ames test showed that GF and AGP were not genotoxic against *Salmonella typhimurium* strains TA98 and TA100, with and without metabolic activation. The present study demonstrated good in vitro cellular compatibility of GF and AGP and. Among these, AGP was the best material as it did not interfere, at any of the tested concentrations, with cell viability and proliferation for up to 72 h of incubation. In any case, neither material induced alterations to cell morphology and were not mutagenic.

## 1. Introduction

The material called graphene, obtained by the micromechanical cleavage of graphite [1], is an atomically thick sheet of sp^2^-hybridized carbon atoms forming a flat honeycomb structure [2]. Applications of graphene are influenced by its hydrophobicity and, therefore, by the impossibility of obtaining stable dispersions in polar solvents [3]. This problem has been overcome by introducing oxygen-containing functional groups in the graphene structure to obtain graphene oxide (GO) [4]. GO shows reduced thermal stability in comparison to the unmodified material but can interact with polar solvents or polymeric matrices, and for these reasons is the most-studied graphene form in biological research. The modification of both graphene and GO make it possible to obtain graphene-based materials (GBMs) which possess suitable properties for specific applications [5,6,7,8]. Graphene flake is of the most popular forms of graphene, and is used in conductive inks, nanofluids, supercapacitors, composites, and hybrid materials with practical utility in a wide range of applications [9]; it can also be obtained in the form of a stable dispersion [10].

Graphene and its derivatives could have a very important role among the new materials being studied thanks to their interesting characteristics that make them excellent candidates for use also as biomaterials [11]. The possible biomedical applications of graphene-based nanomaterials have aroused the interest of many research groups around the world, and thanks to their excellent solubility and selectivity as well as low production costs, they have shown great potential as biosensing and bioimaging materials. Moreover, as they also have unique chemical-physical properties (e.g., high purity, easy solubility, high drug-carrying capacity, easy penetration of cell membranes) they are promising candidates for the construction of bio-delivery carriers [11], but also have potential for use in the field of tissue engineering [12]. To be used as a biomaterial, graphene must meet stringent biocompatibility requirements. In this regard, numerous studies have been conducted on different available forms of graphene. For example, the biocompatibility of graphene flakes and thin graphene films was tested on the human lung epithelial cell line A549. No major changes in morphology or cell adhesion were observed in these cells exposed to graphene flakes in the concentration range 0.1–5 μg/mL for incubation times from 4 to 72 h. Moreover, graphene treatments induced a very slight increase in the cellular production of reactive oxygen species in A549 cells, but no relevant damage to the nuclei (e.g., changes in morphology, condensation, or fragmentation) was observed [13].

Studies performed in mouse embryonic fibroblasts (PMEFs) suggested that biologically synthesized graphene (using a spinach leaf extract) was biocompatible with this cell line, even at doses above 100 μg/mL [14].

However, it is important to underline that while most in vitro studies on graphene have not shown toxicity, others have indicated that GBMs can become hazardous to health [15]. Graphene can exist in different forms, and the degree of biocompatibility can vary depending on the type of material being considered. For example, a study by Bianco et al. pointed out that the toxic response to graphene in vitro and in vivo depends on extrinsic properties such as surface chemistry [16].

Singh et al. showed that GO can induce platelet aggregation comparable to that induced by thrombin, while reduced GO is less effective in inducing this phenomenon [17]. In another study, graphene functionalized with amines did not induce any pro-thrombotic activity, suggesting that it is safer than GO for biomedical applications [18]. The three materials described in this study (i.e., GO, reduced GO, amine-functionalized graphene) differ only in surface chemistry by the presence or absence of oxides and other functional groups. These results emphasize the critical relationship between the surface chemistry of GBMs and their toxicity. However, these surface features are not intrinsic properties of pure graphene but are introduced during manufacturing processes. Additionally, synthesis techniques such as liquid-phase exfoliation and chemical oxidation often require toxic organic solvents, surfactants, strong acids, and oxidants for the exfoliation of graphite flakes. Organic molecules and inorganic impurities that are retained in graphene end products can interact with biological cells and tissues, inducing toxicity or eventually causing cell death. Residual contaminants may cause an increased risk of graphene-induced toxicity in biological cells [19]. Hence, the toxicity of GBMs may not be indicative of the toxicity of pure graphene [13].

In this regard, this study aimed to investigate the degree of in vitro biocompatibility, in terms of cytotoxicity and mutagenicity, of multilayer commercial-grade ultrapure, defect-free, un-oxidized and highly ordered, highly crystalline graphene flakes (GFs) and their stable dispersion, aqueous graphene paste (AGP), in order to assess their possible application as biomaterials. The novelty of this paper is due to the procedures used for the evaluation of in vitro cytotoxicity and genotoxicity. In fact, the experiments reported were performed following the International Standard ISO 10993 (Biological evaluation of medical devices), whose primary aim is the protection of humans from potential biological risks arising from the use of medical devices [20]. Finally, the antitumoral activity of both GF and AGP towards adherent cells and cells in suspension, namely, human breast adenocarcinoma cells (MDA-MB-231) and human monocytes from histiocytic lymphoma (U-937), was also investigated.

## 2. Materials and Methods

### 2.1. Materials

Thrombin, RPMI medium, Dulbecco’s Modified Eagle’s Medium (DMEM), trypsin solution, and all the solvents used for cell culture were purchased from Sigma-Aldrich (Hamburg, Germany). Mouse immortalized fibroblasts (NIH3T3), human breast adenocarcinoma cells (MDA-MB-231), and human monocytes from histiocytic lymphoma (U-937) were from American Type Culture Collection (Manassas, VA, USA). Ames test kit was supplied from Xenometrix (Allschwil, Switzerland).

### 2.2. Graphene Samples

Graphene in two different forms, graphene flake (GF) and aqueous graphene paste (AGP, a stable dispersion of graphene flakes), was provided by Nano Graphene Inc. (Brooklyn, NY, USA). The products were made from multilayer commercial-grade ultrapure, defect-free, un-oxidized, and highly ordered graphene. Highly crystalline graphene flakes were produced using chemical-free, green, patented technology. The characteristics of the two types of products are shown in Table 1.

### 2.3. Methods

#### 2.3.1. In Vitro Cytotoxicity and Antitumoral Activity Evaluation

To evaluate both the in vitro cytotoxicity and antitumor effect of the graphene samples, the direct contact test was used. This test is suitable for samples with various shapes, sizes, and physical statuses (i.e., liquid or solid).

#### 2.3.2. Cell Line for Cytotoxicity Evaluation

The evaluation of in vitro acute toxicity does not depend on the final use for which the product is intended, and the document ISO 10995-5:2009 (Biological evaluation of medical devices—Part 5: Tests for in vitro cytotoxicity) [21] recommends some cell lines from American Type Collection as models to screen the cytotoxicity of novel materials. Among them, I chose NIH3T3 mouse fibroblasts to test graphene’s in vitro cytotoxicity.

#### 2.3.3. Cell Lines for Antitumor Activity Evaluation

In order to evaluate the potential antitumor effect of GF and AGP on both adherent cells and cells growing in suspension, human breast adenocarcinoma cells (MDA-MB-231) and human monocytes from histiocytic lymphoma (U-937) were respectively selected.

#### 2.3.4. Cell Cultures and Evaluation of Cell Viability

NIH3T3 and MDA-MB-231 cells were propagated in DMEM, and U-937 cells in RPMI medium. Both culture media were supplemented with 10% (*v*/*v*) fetal calf serum, 4 mM L-glutamine, 100 IU/mL penicillin, 100 µg/mL streptomycin, and 1% (*v*/*v*) MEM non-essential amino acid solution. NIH3T3, MDA-MB-231, and U-937 cells were incubated at 37 °C in a humidified atmosphere containing 5% CO_2_. Once at confluence, NIH3T3 and MDA-MB-231 cells were washed with 0.1 M PBS, separated with trypsin-EDTA solution, and centrifuged at 1200 RCF for 5 min. NIH3T3 and MDA-MB-231 cells re-suspended in complete medium at a density of 1.5 × 10^4^ cells/mL and U-937 cells at a density of 5 × 10^4^ cells/mL were seeded in each well of 24-well plates and incubated at 37 °C in an atmosphere of 5% CO_2_. After 24 h of culture, the test compounds, properly diluted in completed medium, were added to each well. The following concentrations of both graphene samples (i.e., GF and AGP) were tested: 0.1, 0.2, 0.5, 0.8, 1.0, 1.5, 10, 15, and 20 μg/mL (micrograms of graphene always refers to dry weight material) for 24, 48, and 72 h. The experiments were repeated three times and all samples were set up in six replicates. Complete medium was used as negative control. After 24, 48, and 72 h of incubation, cell viability was evaluated by neutral red uptake (NRU) assay using the procedure already reported [22].

#### 2.3.5. Cell Morphology Determination

To evaluate changes in cell morphology as a function of different concentrations of both GF and AGP, NIH3T3 cells were analyzed by an inverted optical microscope (Olympus IX 70) equipped with a Sony CCD-IRIS video camera. Changes of fibroblasts’ shape were evaluated by Image-J software (National Institute of Health, NIH). Following image acquisition, the two-dimensional conformation of single cells was digitally characterized using two parameters describing shape that are invariant to both size and orientation. Firstly, cell elongation was established by calculating the ratio between the major and minor axes of the best-fitting ellipse of the cell profile. According to Dunn and Heath [23], the elongation represents the numerical value required to minimize the extension of a particular profile, and therefore may provide a quantitative parameter for assessing cell-morphological responses to test materials. Optical images were processed using Image-J software whereby the best fitting ellipse was obtained representing the uniform distribution of the exact number of pixels enclosed by the cell perimeter. Following the establishment of the ellipses’ axes (a and b), the elongation (dimensionless) was calculated by: E = log_2_ (a/b). The second shape descriptor used, circularity, was also determined using Image-J software. The circularity parameter describes the characteristics of a certain geometrical shape relative to that of a circle. It assumes the value of 1 for a perfect circle and decreases, progressively, as the shape become more elongated. The dimensionless circularity of cell shapes was computed using the equation: C = 4 π(area)/(perimeter)^2^. For both elongation and circularity, only single cells were analyzed excluding cell–cell contacting and on-edge objects. A minimum of 50 cells from five different regions in each of six biological repeats was analyzed. Cells were manually traced for outlines. All parameters were measured in micrometers.

#### 2.3.6. Mutagenicity Assay: Ames Test

The TA100 and TA98 strains of *Salmonella typhimurium* were utilized for mutagenicity assay in the absence and presence of metabolic activation (i.e., with and without the S9 fraction). The test strains used were selected because they are sensitive for the detection of a large proportion of known bacterial mutagens and are therefore the most used in the characterization of new materials as recommended in ISO 10993-3:2014 (Biological evaluation of medical devices. Part 3: Tests for mutagenicity, carcinogenicity, and reproductive toxicity) [24]. A specific positive control was used with and without the S9 fraction. 2-Nitrofluorene (2-NF) 50 µg/mL + 4-nitroquinoline N-oxide (4-NQO) 2.5 µg/mL were used as positive controls without metabolic activation, and 2-aminoanthracene (2-AA) was used as a positive control with metabolic activation, respectively. Approximately 10^7^ bacteria were exposed to a range of graphene concentrations (0.1, 0.5, 0.8, 1.0, 20.0, and 10000.0 μg/mL (micrograms of graphene always refers to dry weight material)), and to positive and negative controls, for 90 min in medium containing sufficient histidine to support approximately two cell divisions. After 90 min, the exposure cultures were diluted in pH indicator medium lacking histidine, and aliquoted into 48 wells of a 384-well plate. Within two days, cells which had undergone the reversion to histidine grew into colonies. The metabolism of the bacterial colonies reduced the pH of the medium, changing the color of that well. This color change could be detected visually. The number of wells containing revertant colonies was counted for each dose and compared to a zero-dose control. Each dose was tested in six replicates. The material was defined as mutagenic if the number of histidine revertant colonies was twice or more than the spontaneous revertant ones.

#### 2.3.7. Statistical Analysis

All assays were carried out three times in six replicates, and results were expressed as mean ± standard deviation (SD). Multiple comparisons were performed by one-way ANOVA and individual differences tested by Fisher’s test after the demonstration of significant intergroup differences by ANOVA. Differences with *p* < 0.05 were considered significant.

## 3. Results

### 3.1. In Vitro Cytotoxicity: Cell Viability, Proliferation, and Morphology

#### 3.1.1. Cell Viability and Proliferation

**Figure 1:**
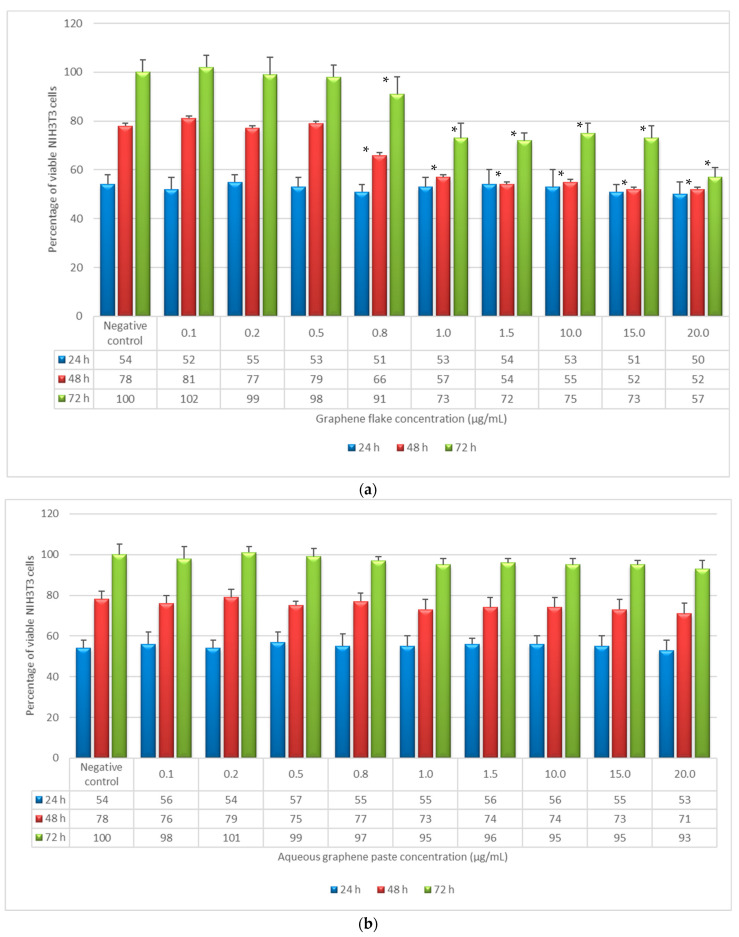
Percentage of viable NIH3T3 cells as a function of incubation time (24, 48, and 72 h) and sample concentration, as determined by the neutral red uptake: (**a**) NIH3T3 + graphene flake; (**b**) NIH3T3 + aqueous graphene paste. Data are mean ± SD of three experiments run in six replicates. * Values are statistically different versus negative control (complete medium), *p* < 0.05.

Non-confluent adherent mouse fibroblasts (NIH3T3) were incubated with different concentrations of GF and AGP. Cells were analyzed after 24, 48, and 72 h of contact with the test samples, and the results in terms of cell viability (%) as a function of both samples’ concentration and incubation time are reported in Figure 1.

The data reported in Figure 1a show that after 24 h of incubation the percentage of viable cells was not statistically different in comparison to the negative control for all tested GF concentrations. In contrast, the percentage of viable cells in the presence of GF decreased compared to the negative control for concentrations from 0.8 to 20 μg/mL and incubation times of 48 and 72 h, but only the highest concentration reduced the percentage of viable cells by more than 30% in comparison to the negative control. Regarding cytotoxicity, the standard states that a material can be considered non-cytotoxic if it allows cell viability greater than 70% after exposure for 24 h. In this regard, GF cannot be considered cytotoxic at any of the analyzed concentrations.

AGP was unable to influence the viability and proliferation of mouse fibroblasts at any of the tested concentrations and incubation times, as shown in Figure 1b.

However, neither of the graphene materials (i.e., GF and AGP) affected the cytotoxicity in terms of cell viability and proliferation after 24 h, and this is a requirement of ISO standards to define a material as non-cytotoxic.

#### 3.1.2. NIH3T3 Morphology

**Figure 2:**
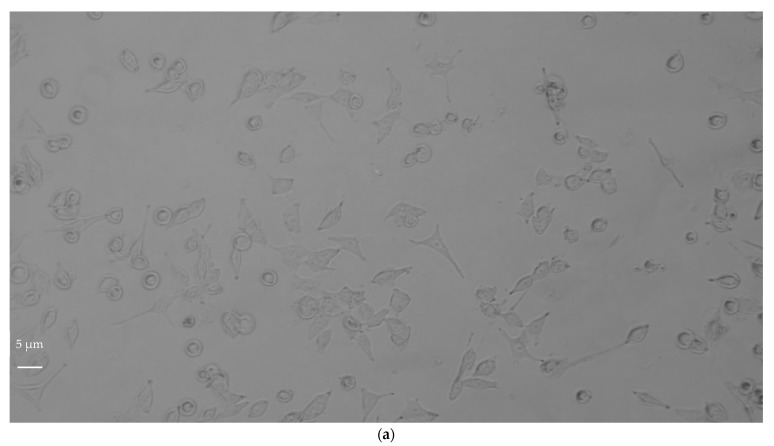

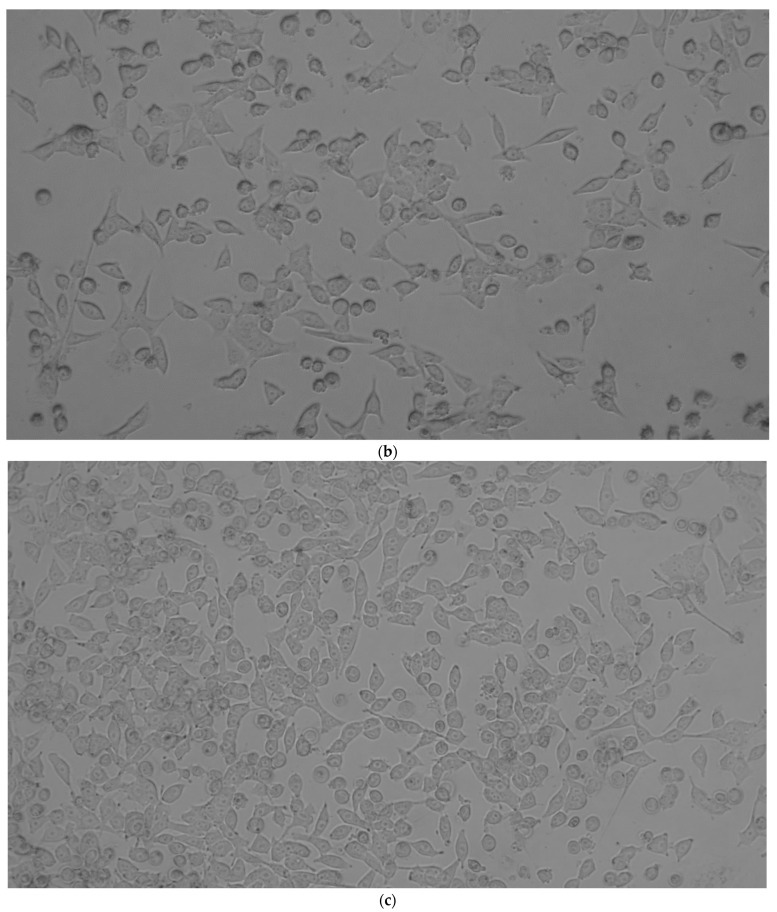

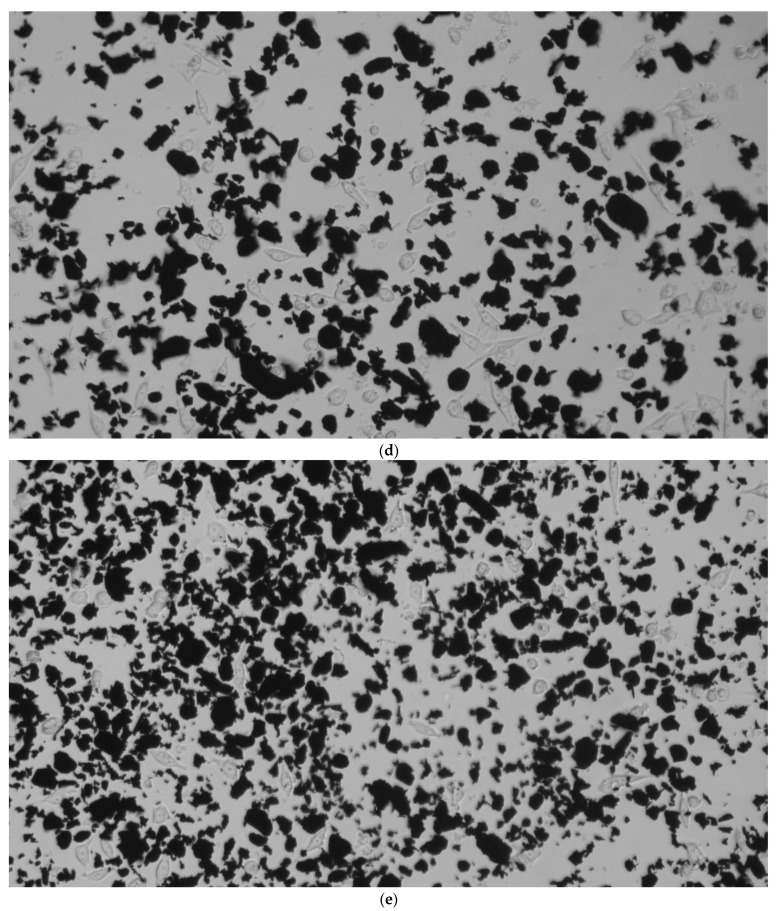

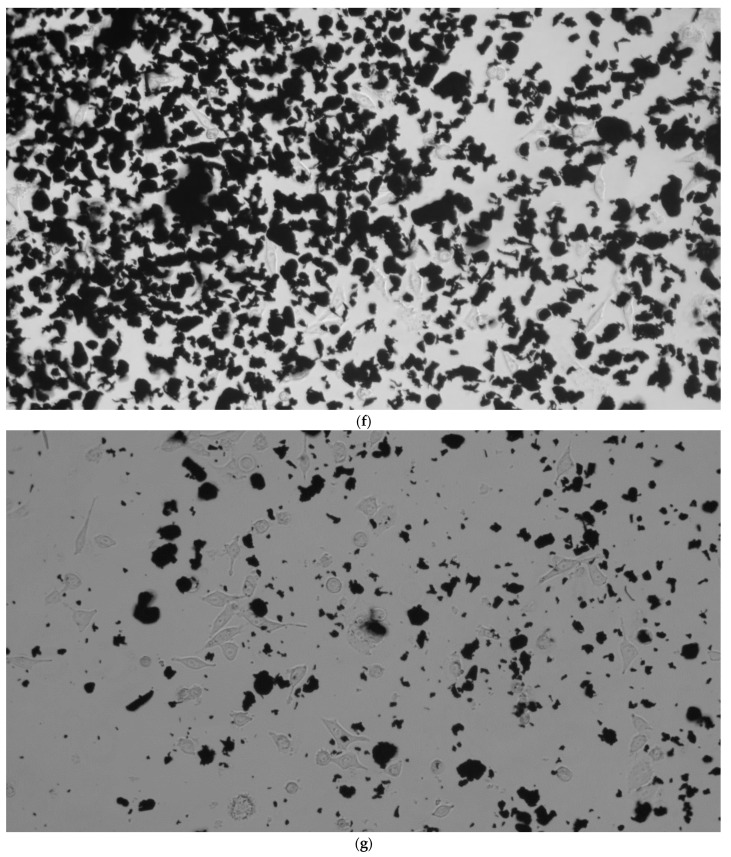

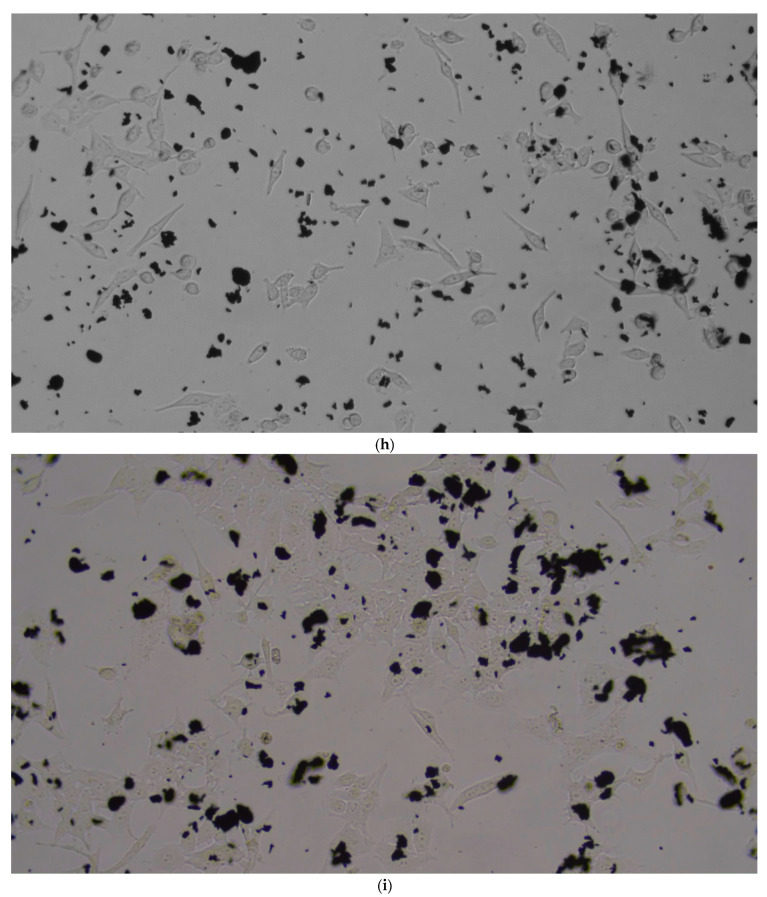
Optical microscopy images of NIH3T3 cells as a function of incubation time at the highest concentration of GF and AGP tested (20 µg/mL). Magnification 20×, scale bar = 5 µm. (**a**) Negative control, 24 h; (**b**) negative control, 48 h; (**c**) negative control, 72 h; (**d**) GF, 24 h; (**e**) GF, 48 h; (**f**) GF, 72 h; (**g**) AGP, 24 h; (**h**) AGP, 48 h; (**i**) AGP, 72 h.

The qualitative evaluation of a material’s cytotoxicity consists of the examination of cells by optical microscopy for assessing changes in general morphology, vacuolization, detachment, cell lysis, and membrane integrity [21]. NIH3T3 fibroblasts are bipolar or multipolar cells with an elongated shape which grow attached to a substrate. Changes in NIH3T3 morphology, such as decreased elongation and increased circularity, after exposure to materials is correlated to the ability of the material to interfere with cell viability. After 24, 48, and 72 h of incubation with increasing concentrations of both GF and AGP, separately, NIH3T3 cells showed no modifications in cell elongation or circularity (Table 2a,b). These data are also confirmed by the light-microscopy images shown in Figure 2 and Figure S1. The cells in contact with GF showed no relevant damage to the nuclei, such as changes in morphology, condensation, or fragmentation, and maintained fibroblastic morphology for all incubation times and all concentrations tested; however, at the highest concentrations (20 µg/mL) the cell density decreased with increasing incubation time (Figure 2a). At the exposure concentration of 20 μg/mL the cells were found to be completely covered with nanomaterial aggregates. This could cause graphene-unspecific cell damage due to mechanical stress, and thus may have affected cell density as revealed by the quantitative cytotoxicity evaluation reported in Figure 1a.

NIH3T3 cells in contact with AGP (Figure 2b) showed bipolar or multipolar shape, and cell morphology and density were not affected by either sample concentration or incubation time. At the exposure concentration of 20 μg/mL, the few nanomaterial aggregates present were not able to induce graphene-unspecific cell damage due to mechanical stress, or to affect cell density, as demonstrated by the quantitative cytotoxicity evaluation reported in Figure 1b.

### 3.2. Antitumor Activity

**Figure 3:**
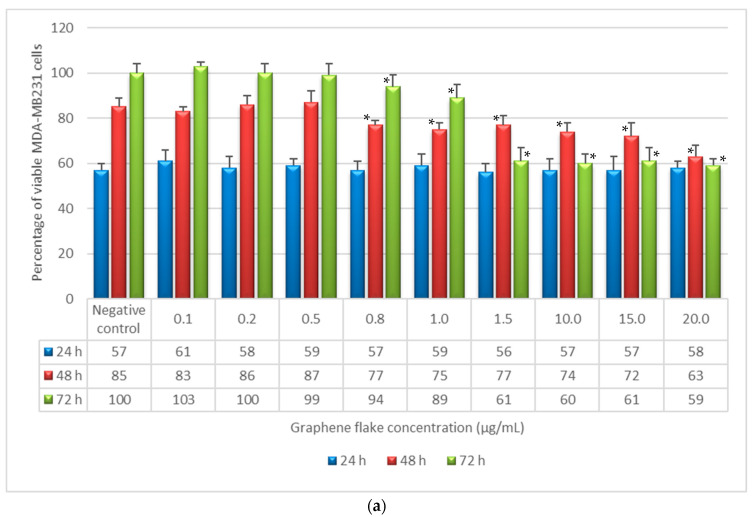

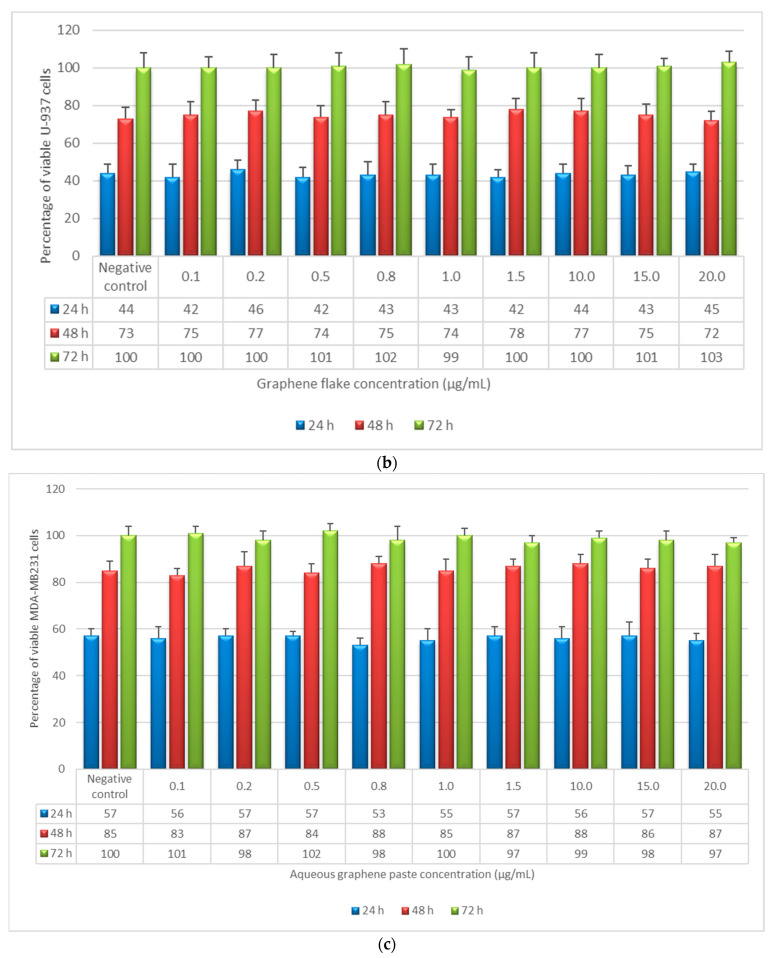

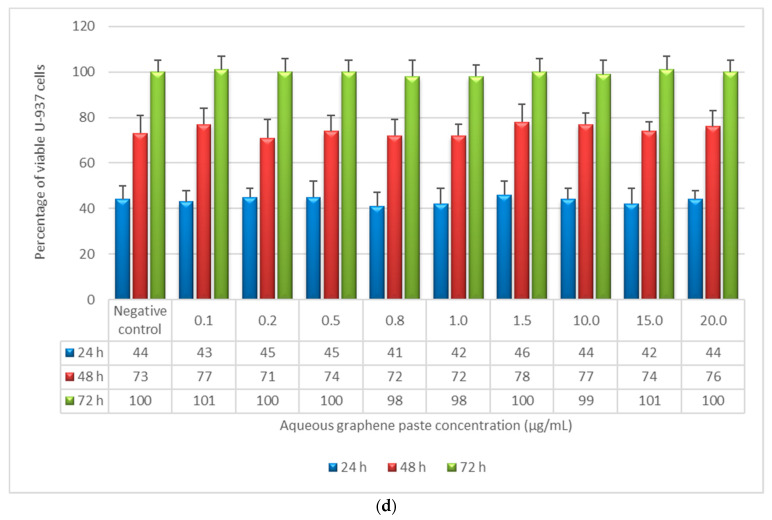
Percentage of viable MDA-MB-231 and U-937 as a function of incubation time (24, 48, and 72 h) and sample concentration, as determined by the neutral red uptake assay: (**a**) MDA-MB-231 + graphene flake; (**b**) U-937 + graphene flake; (**c**) MDI-MB-231 + aqueous graphene paste; (**d**) U-937 + aqueous graphene paste. Data are mean ± SD of three experiments run in six replicates. * Values are statistically different versus negative control (complete medium), *p* < 0.05.

The antitumor activity of GF and AGP was also evaluated by NRU assay on both human breast adenocarcinoma cells (MDA-MB-231) and human monocytes from histiocytic lymphoma (U-937). Results in terms of cell viability (%) as a function of sample concentration and incubation time are reported in Figure 3.

The data in Figure 3a show that the percentage of MDA-MB-231 cells in contact with GF increased with increasing incubation time in a way that was not significantly different with respect to negative control, for concentrations between 0.1 and 0.5 µg/mL. In contrast, GF reduced the percentage of viable cells compared to the negative control for concentrations from 0.8 to 20 µg/mL and incubation times of 48 and 72 h. The most toxic concentration was found to be 20 µg/mL after 72 h of incubation, which led to a reduction in the percentage of viable MDA-MB-231 cells of about 40% in comparison to the negative control.

U-937 cell viability and proliferation were not significantly affected by contact with any GF concentrations tested for all three incubation times, as shown in Figure 3b.

AGP was demonstrated to have no toxic effect, in terms of cell viability and proliferation, towards both adenocarcinoma breast cells and monocytes at all incubation times and for all concentrations tested (Figure 3c,d).

### 3.3. Mutagenicity Assay: Ames Test

**Figure 4:**
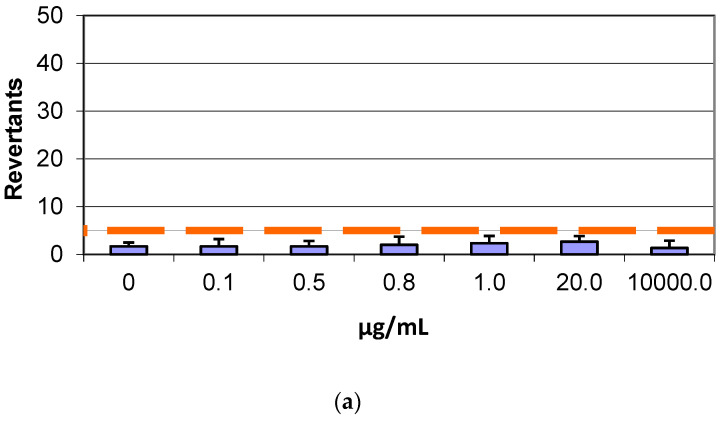

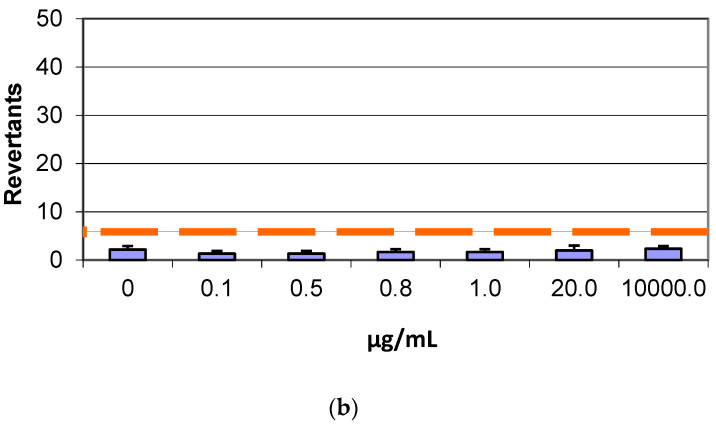

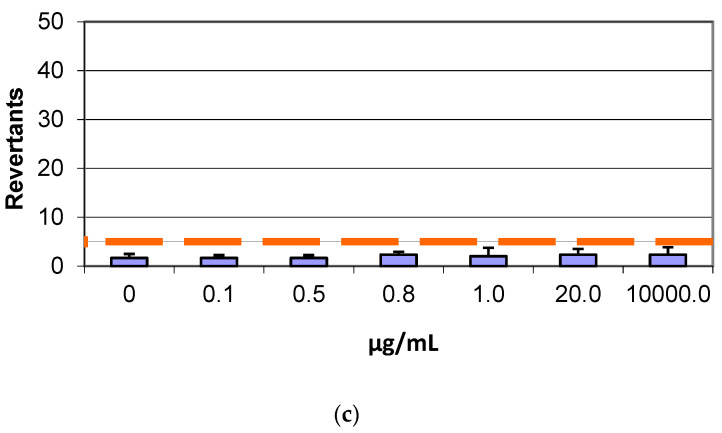

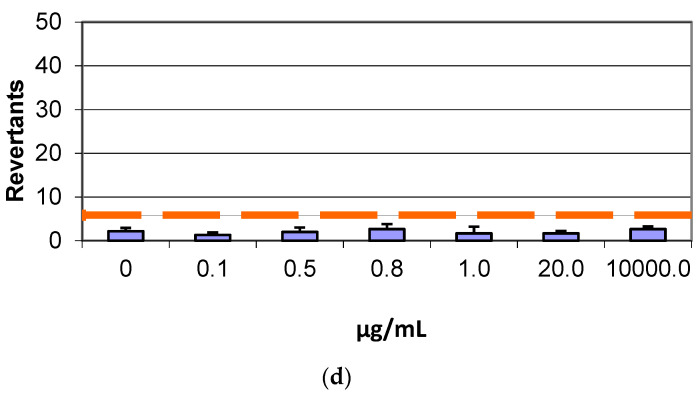
Number of revertants in TA98 *S. typhimurium* strain exposed to different concentrations of (**a**) GF without S9 fraction; (**b**) GF with S9 fraction; (**c**) AGP without S9 fraction; (**d**) AGP with S9 fraction. The results are reported as the mean of revertants ± SD, *p* < 0.05.

**Figure 5:**
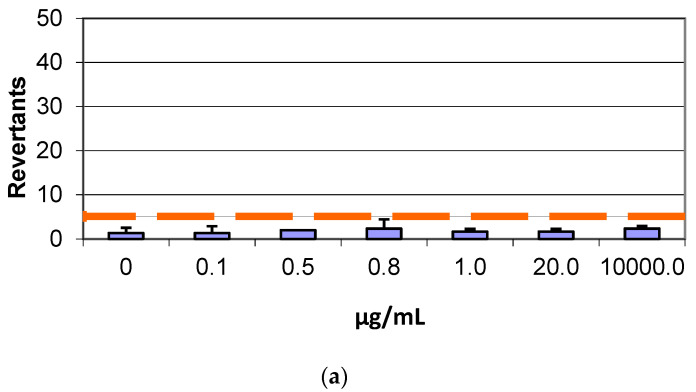

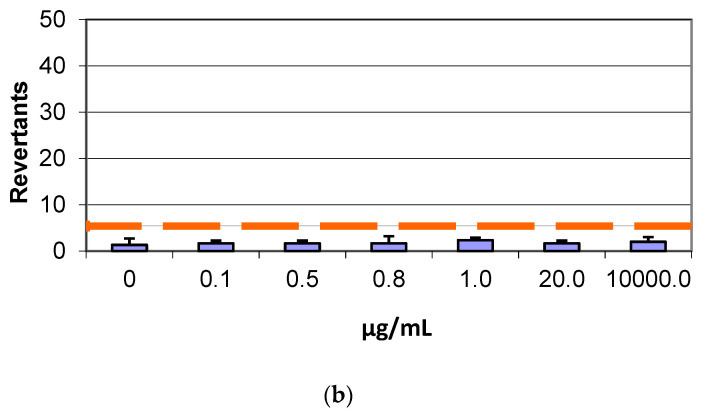

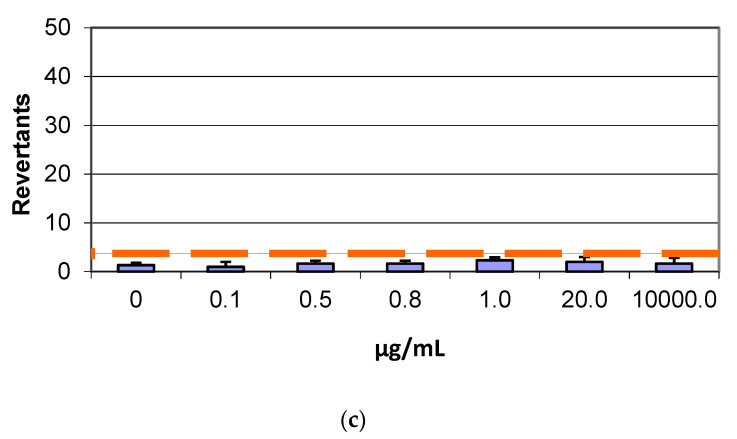

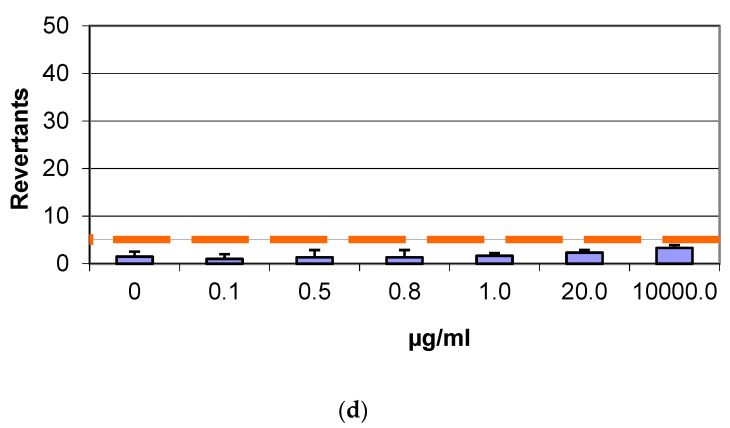
Number of revertants in TA100 *S. typhimurium* strain exposed to different concentrations of (**a**) GF without S9 fraction; (**b**) GF with S9 fraction; (**c**) AGP without S9 fraction; (**d**) AGP with S9 fraction. The results are reported as the mean of revertants ± SD, *p* < 0.05.

In the Salmonella mutagenicity assay, six different concentrations of both GF and AGP, separately, were tested on TA98 and TA100 strains with and without S9 metabolic activation. The results for the genotoxic effect reported in Figure 4 and Figure 5 showed no increase of revertants per plate with increasing concentrations of GF and AGP for both TA98 and TA100 with and without S9 fraction, demonstrating the lack of dose–response genotoxicity in the samples. In fact, at the highest graphene concentration tested (10000 µg/mL), the number of revertants was lower and statistically different in comparison to the positive control (*p* < 0.05). The background level and positive control values were in all cases within the normal limit found in our laboratory and in accordance with literature data [25].

## 4. Discussion

The success of a biomaterial is based on the understanding of the cell–material interactions, which above all must not alter cell viability and function. The present findings on the preliminary in vitro assessment of the biocompatibility of two different ultrapure graphene samples (GF and AGP) towards NIH3T3 mouse fibroblasts demonstrated that they are biocompatible and not cytotoxic to these cells. However, the observed differences between samples in terms of cell viability and proliferation as a function of both sample concentration and incubation time indicate that GF and AGP are biocompatible but to different extents. AGP did not affect cell viability and proliferation at any of the tested concentrations for all incubation times (24, 48, and 72 h). In contrast, cell proliferation was reduced in the presence of GF concentrations from 0.8 to 20 µg/mL and incubation times of 48 and 72 h. The differences in the biocompatibility degree between GF and AGP may be due to the different compositions of the two specimens in terms of graphene content. In fact, as reported in Table 1, the percentage of graphene in AGP was about 55–60%. Therefore, at the same concentration, the GF sample contained a greater quantity of graphene than AGP, and this likely influenced the characteristics of cytotoxicity in terms of cell viability and proliferation for different reasons. First, the GF aggregates formed (Figure 2d–f and Figure S2a) were more numerous than those of AGP (Figure 2g–I and Figure S2b) and therefore covered a greater surface area, inducing mechanical stress on the underlying cells and limiting the free surface area available for cell adhesion. In support of this, the cell density decreased with increasing GF concentration but for incubation times longer than 24 h (Figure 1a). These data agree with the study of Lammel et al. [26] which demonstrated that cells exposed to graphene oxide (GO) and carboxyl graphene (CXYG) concentrations of 16 μg/mL for 24 h were completely covered with nanomaterial and this may have caused nanomaterial-unspecific cell damage due to mechanical stress, increased the probability of interference of the nanomaterial with the assay, or both. Moreover, Coleman et al. [27] observed size-, concentration-, and time-dependent cytotoxicity of single-layered GO on an NIH3T3 cell line, and experiments performed on in-house-prepared GO processed via base washing, sonication, cleaning, and combinations demonstrated that the morphology of graphene samples also greatly impacted their cytotoxicity [28]. Small-flaked GO stimulated the proliferation of vascular smooth muscle cells, suggesting the potential of using the surface chemistry or physical properties of GO to influence cell growth behavior [29].

**Figure 6:**
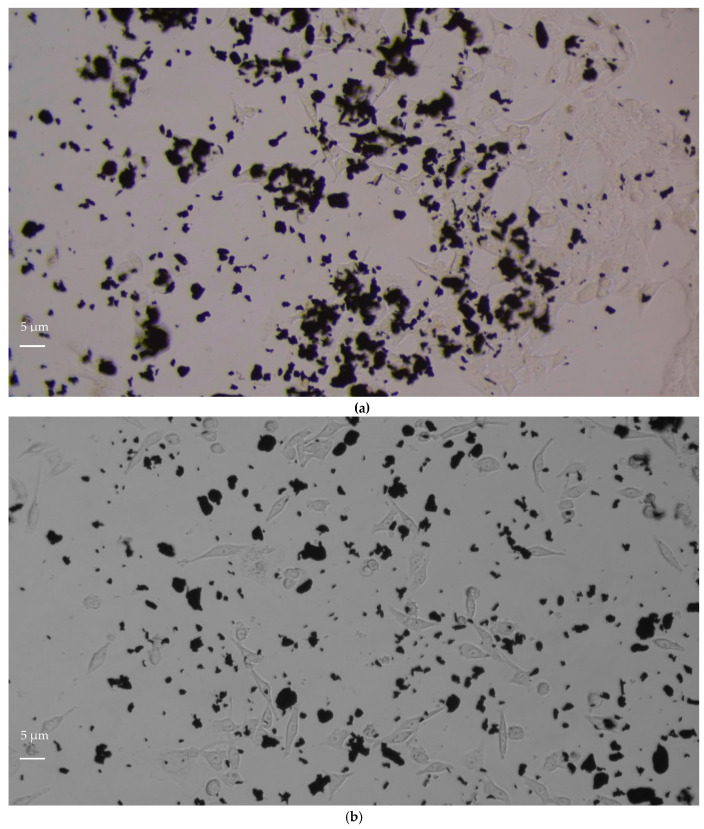
Optical microscopy images of NIH3T3 cells after 24 h of incubation with: (**a**) GF 10 µg/mL; (**b**) AGP 20 µg/mL. Magnification 20×, scale bar = 5 µm.

AGP and GF at 20 µg/mL and 10 µg/mL respectively possessed the same graphene concentration but differently influenced cell viability, as shown in Figure 1. In particular, the percentage of viable fibroblasts decreased for incubation times of 48 and 72 h in the presence of GF but not with AGP. This different behavior between the two graphene samples may have been due to the different size and morphology of graphene aggregates. As shown in Table 3 and Figure 6a, GF at a concentration of 10 µg/mL showed larger aggregates (66% with dimensions of about 10 µm x 4.5 µm) in comparison to AGP at 20 µg/mL (63% with dimensions of 1.4 µm x 0.9 µm). This trend agrees with observations reported by Ren et al. [29] regarding VSMCs growth, which was stimulated by smaller flakes, but disagrees with the observation of Wychowaniec et al. [30] which demonstrated a higher cytotoxicity of smaller GO flakes.

The potential mechanical influence of GF aggregates on reducing cell viability and proliferation was also investigated in MDA-MB-231 and U-937 cell lines, in adherent cells and cells in suspension, respectively. Adherent MDA-MB-231 cells showed the same trend as NIH3T3 cells in terms of toxicity. In fact, GF reduced the percentage viable cells compared to the negative control for the highest concentrations and incubation times of 48 and 72 h. The most toxic concentration was found to be 20 µg/mL after 72 h of incubation, which led to a reduction in the percentage of viable MDA-MB-231 cells of about 40% in comparison to the negative control (Figure 3a). In contrast, U-937 cell viability and proliferation in contact with all GF concentrations and for all the three incubation times was not affected by the samples, as shown in Figure 3b. Finally, AGP was demonstrated to have no toxic effect on either adenocarcinoma breast cells or monocytes at all incubation times and for all concentrations as shown in Figure 3c,d. Because U-937 cells are grown in suspension, they were not affected by the presence of GF aggregates and their viability and proliferation were not decreased, even at the highest GF concentrations. On the contrary, as MDA-MB-231 are adherent cells, and their viability and proliferation were affected by the highest concentrations of GF and larger graphene aggregates, as was also observed for NIH3T3 cells. Therefore, neither GF nor AGP showed any specific antitumor activity, and the decrease in cell viability and proliferation observed for MDA-MB-231 cells exposed to the highest GF concentrations could be due to a mechanical effect of GF agglomerates interfering with cell adhesion. Although the mechanical stress due to GF aggregates may play an important role, a dose-dependent toxicological effect involved in the reduction of cell viability and proliferation cannot be excluded, which was also observed for some multi-walled carbon nanotubes [31], and for the cytotoxicity in Hep G2 cells with plasma membrane damage induced by GO and CXYG nanoplatelet suspensions [26]. Moreover, Nasirzadeh et al. [32] demonstrated that the toxicity of graphene nanoparticles (GNPs) on A549 epithelial cells of the human lung was time-dependent, and the GNPs were more cytotoxic after 72 h of incubation compared to at 24 and 48 h, with a half-maximal inhibitory concentration of 40 µg/mL [33]. These results agree with the demonstration that GF at the highest concentration (20 µg/mL) reduced the viability of NIH3T3 cells by 43% after 72 h of exposure. Interactions of graphene with cell membranes can also induce membrane damage and cell death. Cell membranes contain phospholipids, which consist of a phosphate head group and two fatty acid chains; and cholesterol molecules, which play important roles in maintaining membrane physiology. Pure graphene with no charges on the basal plane cannot interact electrostatically with phospholipids but is able to promote hydrophobic interactions with cholesterol tails, which can lead to the extraction or removal of cholesterol molecules from the membrane, leading to membrane deformation and damage, as well as alteration of the physiological cell morphology [33]. Therefore, cell morphology is the main indicator of this phenomenon and expresses the status of cells. Morphological alterations were not observed in NIH3T3 fibroblasts following exposure to GF and AGP. Neither the graphene-treated nor the control cells showed any differences in their adhesion to the culture medium dish. The NIH3T3 cells maintained their bipolar or multipolar elongated shape and adhered to the substrate. Cells in contact with GF maintained fibroblastic morphology for all incubation times and at all concentrations tested, including at the highest values when cell density decreased by increasing the incubation time without evidence of morphological cell alterations. The cause of the cytotoxicity of GF in terms of decreased of cell viability and proliferation cannot be due to its interaction with membrane cholesterol; the most important factors are probably the mechanical stress exerted on the adherent cells and the decrease of the adhesive surface available due to the presence of numerous and large GF agglomerates at the highest tested graphene concentrations.

A genotoxicity study is a key step for risk assessment during the evaluation of materials for human applications because various genotoxic compounds can cause DNA damage, compromising human health [34]. In the bacterial reverse mutation assay (Ames test) performed on GF and AGP, as expected, the positive control chemicals significantly induced mutagenicity. However, no mutagenic positive result was observed in GF- and AGP-treated groups compared to control at any of the tested concentrations. These results indicated that the samples analyzed did not exhibit any mutagenic risk under the experimental conditions of this study.

The lateral size, shape, aggregation state, oxidation state, surface properties, chemistry, concentration, and preparation techniques of graphene-based nanomaterials are all parameters which influence their bioactivity and toxicity—not only in vitro but also in vivo. The results of in vivo toxicity studies in laboratory mammals reported in literature in fact demonstrate that graphene shows potential pulmonary, systemic, behavioral, reproductive, and developmental toxicity and genotoxicity, probably due to induction of oxidative stress and inflammation. Although many acute toxicity studies have been performed, no long-term toxicity studies have been conducted [35]. Therefore, even if graphene is potentially usable in every branch of science and technology, more research is needed to exploit its full potential—especially for its application as a biomaterial.

## 5. Conclusions

The present study demonstrated that both GF and AGP showed good cell compatibility in vitro in terms of both NIH3T3 cell viability and proliferation. The best material was AGP, which did not interfere, at any of the tested concentrations, with cell proliferation following an incubation period of up to 72 h. Neither material induced alterations in cell morphology in terms of elongation and circularity and were not mutagenic.

The antitumor activity experiments demonstrated that MDA-MB-231 cell viability and proliferation had the same trend observed for NIH3T3 cells. GF reduced the percentage of viable cells compared to the negative control for the highest tested concentration values and incubation times of 48 and 72 h. U-937 cell viability and proliferation were not affected by contact with all GF concentrations tested for all three incubation times. AGP was demonstrated to have no toxic effect on either adenocarcinoma breast cells or monocytes at all incubation times and for all tested concentrations.

Although the NRU measurement is a reliable toxicity marker, the test has some inherent limitations. This measurement, which is a colorimetric assay, was used to determine cytotoxicity in terms of cell viability and proliferation without considering the toxicity mechanisms. It is recommended that the determination of oxidative stress, apoptosis, LDH, superoxide dismutase, and autophagy (which can influence cytotoxicity) be considered to better understand the toxicity mechanisms of GF together with graphene aggregate size and morphology as a function of sample concentration.

All these preliminary data indicate that the two graphene formulations evaluated are excellent candidates for further in vitro and in vivo biocompatibility testing to ascertain their biological safety for the realization of new graphene-based medical devices.

## Figures and Tables

**Table 1:** Products features.

Products	Purity (%)	Carbon Content (%)	Oxygen Content (%)
GF	99.8	99.7	<0.25
AGP (graphene content 55–60%)	99.8	99.6	-

**Table 2:** NIH3T3 cell elongation and circularity after 24, 48, and 72 h of contact with different concentrations of GF (a) and AGP (b). No value was statistically different versus negative control (complete medium), *p* < 0.05.

(**a**)						
**GF**						
		**24 h**	**48 h**		**72 h**	
**Concentration** **(µg/mL)**	**Elongation** **(Mean ± SD)**	**Circularity** **(Mean ± SD)**	**Elongation** **(Mean ± SD)**	**Circularity** **(Mean ± SD)**	**Elongation** **(Mean ± SD)**	**Circularity** **(Mean ± SD)**
Control	1.43 ± 0.08	0.369 ± 0.115	1.42 ± 0.06	0.371 ± 0.113	1.43 ± 0.05	0.367 ± 0.119
0.1	1.39 ± 0.07	0.373 ± 0.106	1.41 ± 0.08	0.355 ± 0.132	1.42 ± 0.05	0.358 ± 0.122
0.2	1.40 ± 0.07	0.357 ± 0.105	1.38 ± 0.06	0.362 ± 0.126	1.39 ± 0.07	0.363 ± 0.116
0.5	1.41 ± 0.05	0.367 ± 0.129	1.40 ± 0.09	0.358 ± 0.115	1.39 ± 0.05	0.368 ± 0.111
0.8	1.39 ± 0.06	0.362 ± 0.116	1.42 ± 0.08	0.361 ± 0.113	1.41 ± 0.07	0.359 ± 0.117
1.0	1.41 ± 0.07	0.359 ± 0.119	1.38 ± 0.05	0.356 ± 0.115	1.37 ± 0.07	0.359 ± 0.114
1.5	1.43 ± 0.05	0.361 ± 0.115	1.38 ± 0.07	0.357 ± 0.118	1.39 ± 0.05	0.356 ± 0.121
10	1.42 ± 0.05	0.362 ± 0.103	1.38 ± 0.08	0.359 ± 0.110	1.40 ± 0.04	0.357 ± 0.110
15	1.40 ± 0.07	0.360 ± 0.111	1.39 ± 0.04	0.357 ± 0.119	1.39 ± 0.08	0.357 ± 0.117
20	1.41 ± 0.07	0.358 ± 0.109	1.37 ± 0.06	0.354 ± 0.122	1.38 ± 0.07	0.355 ± 0.119
(**b**)						
**AGP**						
	**24 h**		**48 h**		**72 h**	
**Concentration** **(µg/mL)**	**Elongation** **(Mean ± SD)**	**Circularity** **(Mean ± SD)**	**Elongation** **(Mean ± SD)**	**Circularity** **(Mean ± SD)**	**Elongation** **(Mean ± SD)**	**Circularity** **(Mean ± SD)**
Control	1.43 ± 0.08	0.369 ± 0.115	1.42 ± 0.06	0.371 ± 0.118	1.43 ± 0.05	0.367 ± 0.119
0.1	1.41 ± 0.07	0.367 ± 0.123	1.42 ± 0.05	0.366 ± 0.115	1.44 ± 0.07	0.361 ± 0.121
0.2	1.44 ± 0.06	0.369 ± 0.131	1.44 ± 0.08	0.366 ± 0.118	1.42 ± 0.09	0.355 ± 0.123
0.5	1.43 ± 0.06	0.368 ± 0.122	1.45 ± 0.04	0.363 ± 0.112	1.38 ± 0.04	0.359 ± 0.114
0.8	1.38 ± 0.05	0.364 ± 0.118	1.39 ± 0.07	0.359 ± 0.115	1.41 ± 0.06	0.363 ± 0.111
1.0	1.39 ± 0.07	0.364 ± 0.115	1.38 ± 0.06	0.360 ± 0.114	1.41 ± 0.08	0.361 ± 0.117
1.5	1.42 ± 0.06	0.360 ± 0.113	1.41 ± 0.07	0.363 ± 0.108	1.39 ± 0.05	0.355 ± 0.116
10	1.40 ± 0.08	0.362 ± 0.109	1.39 ± 0.05	0.361 ± 0.112	1.40 ± 0.07	0.360 ± 0.119
15	1.42 ± 0.04	0.361 ± 0.116	1.42 ± 0.06	0.364 ± 0.119	1.41 ± 0.07	0.362 ± 0.117
20	1.40 ± 0.08	0.363 ± 0.119	1.44 ± 0.05	0.366 ± 0.121	1.43 ± 0.09	0.362 ± 0.120

**Table 3:** Size distribution of GF 10 µg /mL and APG 20 µg /mL aggregates in culture medium (DMEM).

GF 10 µg /mL		AGP 20 µg /mL	
Aggregate size (µm^2^)	Percentage	Aggregate size (µm^2^)	Percentage
(10 × 4.5)	66%	(5.0 × 4.5)	4%
(4.5 × 3.2)	23%	(4.5 × 3.6)	5%
(3.2 × 1.8)	4%	(2.7 × 1.8)	7%
(3.2 × 0.5)	6%	(1.4 × 0.9)	63%
(1.8 × 0.5)	1%	(0.5 × 0.2)	21%

## Data Availability

Not applicable.

## Supplementary Materials

The following supporting information can be downloaded at: www.mdpi.com/article/10.3390/life12020242/s1, Figure S1: Optical microscopy images of NIH3T3 cells at 24, 48 and 36 h of contact time with 20 µg/mL GF and AGP after the removal of the graphene samples. (a) GF, 24 h; (b) GF 48 h; (c) GF 72 h; (d) AGP 24 h; (e) AGP, 48 h; (f) AGP, 72 h. Magnification 20×, scale bar= 5 µm. Figure S2: Optical microscopy images of NIH3T3 as a function of incubation time and concentration of Graphene Flake (a) and Aqueous Graphene Paste (b). The density and size of aggregates increase as the concentration of GF and AGP increases. Magnification 20×. Scale bar= 5 µm.
