# Supplementary material for: Preliminary In Vitro Cytotoxicity, Mutagenicity and Antitumoral Activity Evaluation of Graphene Flake and Aqueous Graphene Paste
Source: Life (Basel). 2022 Feb 7;12(2):242. doi: 10.3390/life12020242 (PMC8878666; doi:10.3390/life12020242)
Supplement: Supplementary file 1 [file life-12-00242-s001.zip › life-1554268-supplementary.pdf]

life

MDPI

Supplementary

Preliminary *In Vitro* Cytotoxicity, Mutagenicity and Antitumoral Activity Evaluation of Graphene Flake and Aqueous Graphene Paste

Stefania Lamponi

Department of Biotechnology, Chemistry and Pharmacy and SienabioACTIVE, University of Siena, Via Aldo Moro 2, 53100 Siena, Italy; Stefania.Lamponi@unisi.it; Tel.: +39-0577-232110; Fax: +39-0577-234254

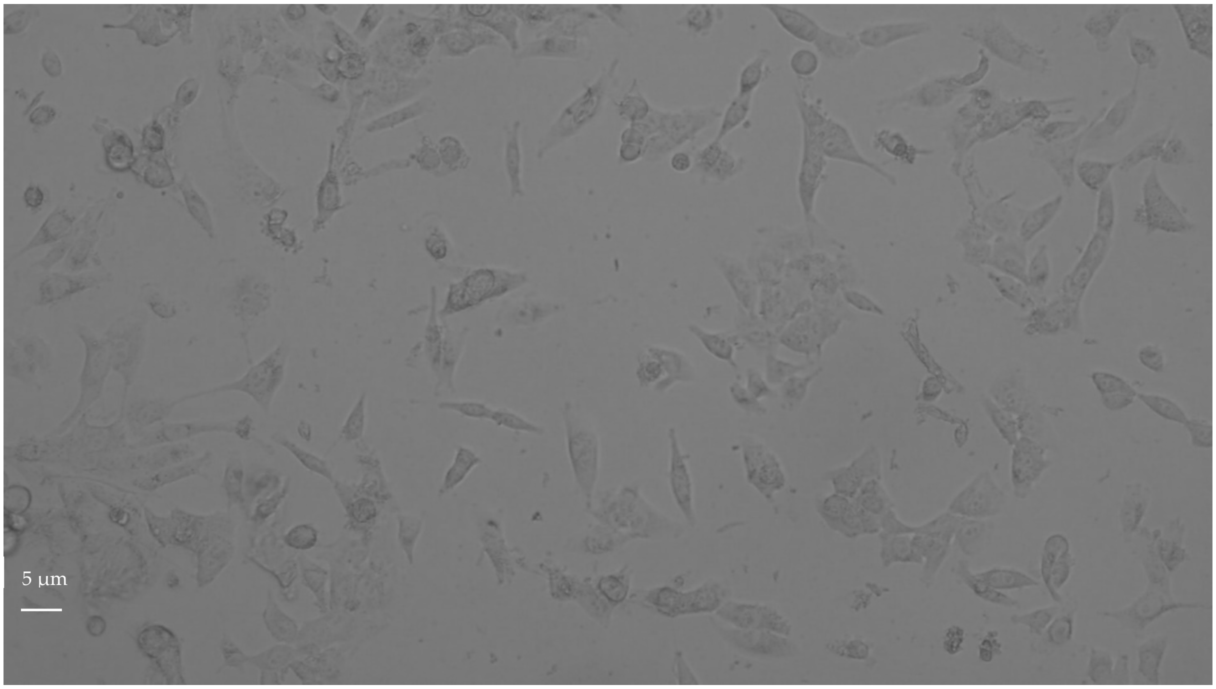

(a)

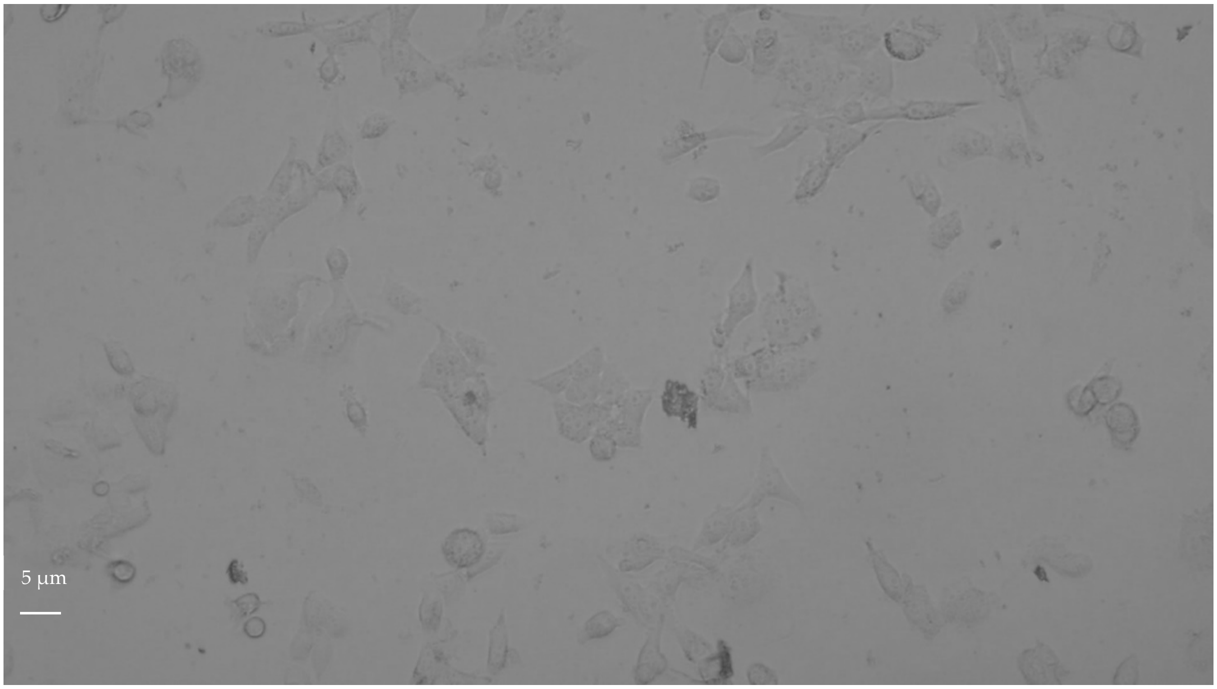

(b)

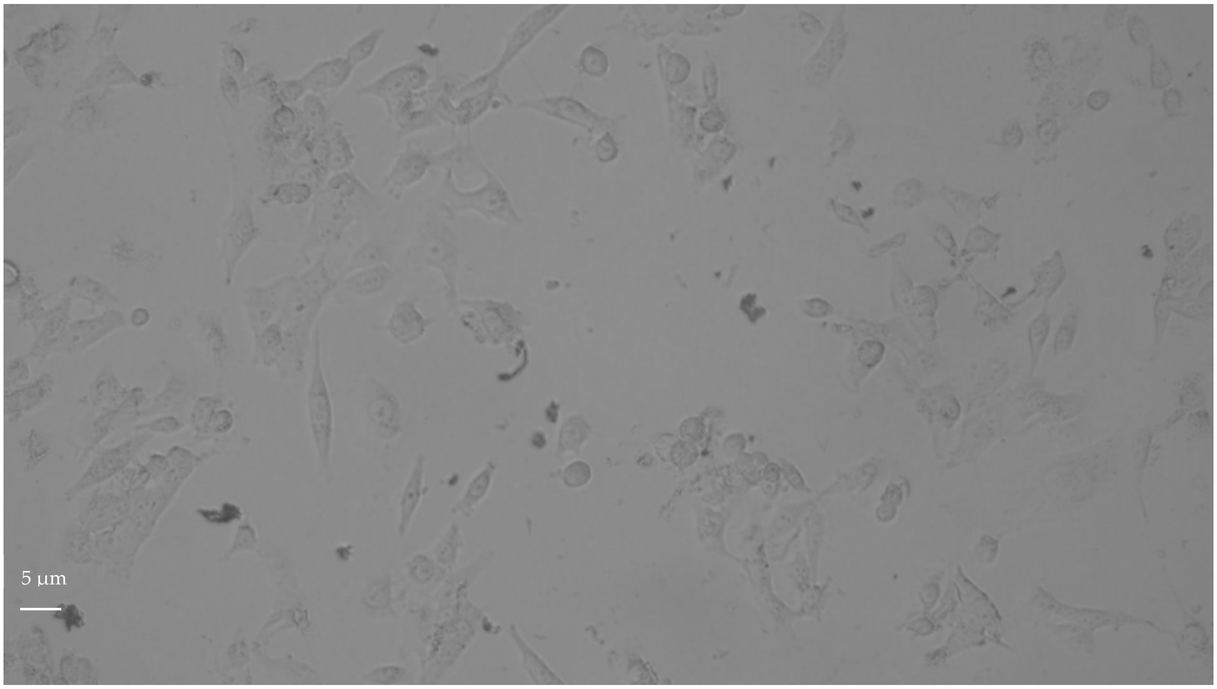

(c)

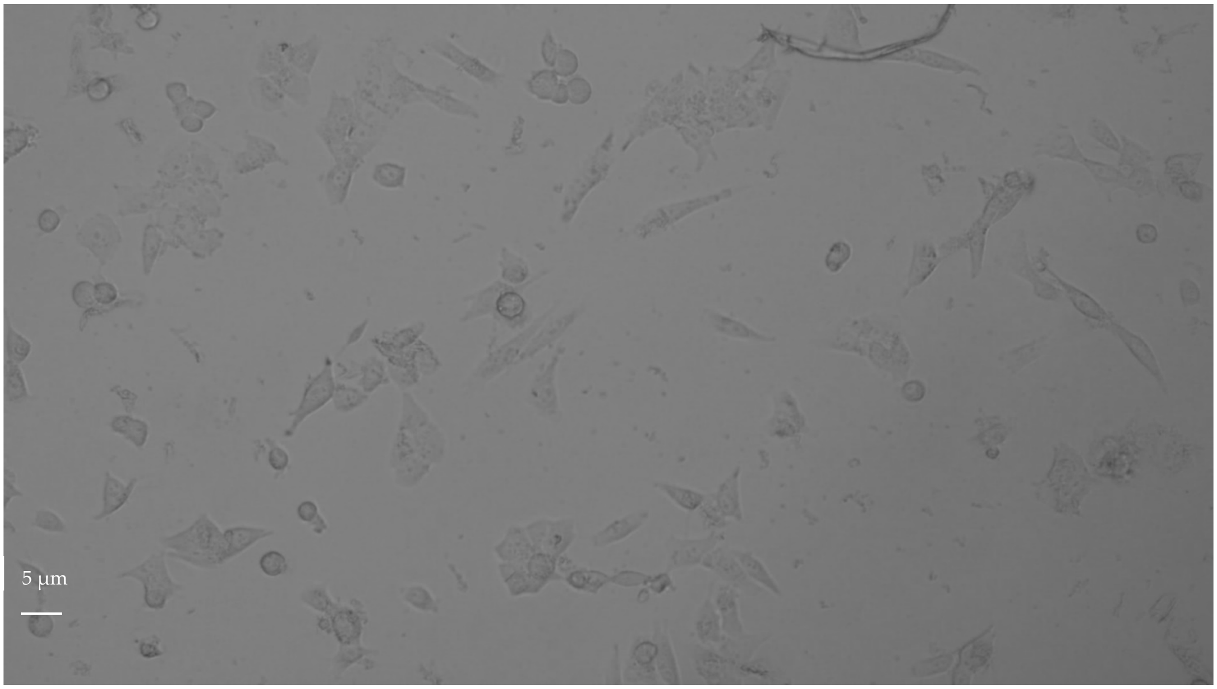

(d)

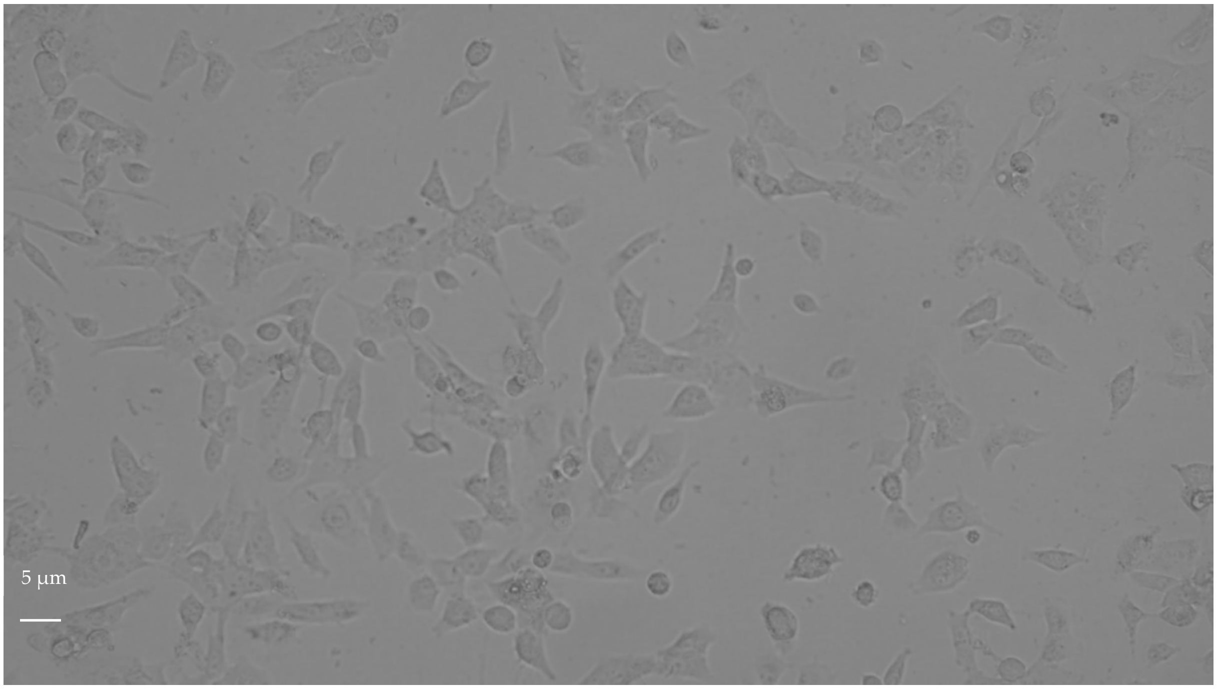

(e)

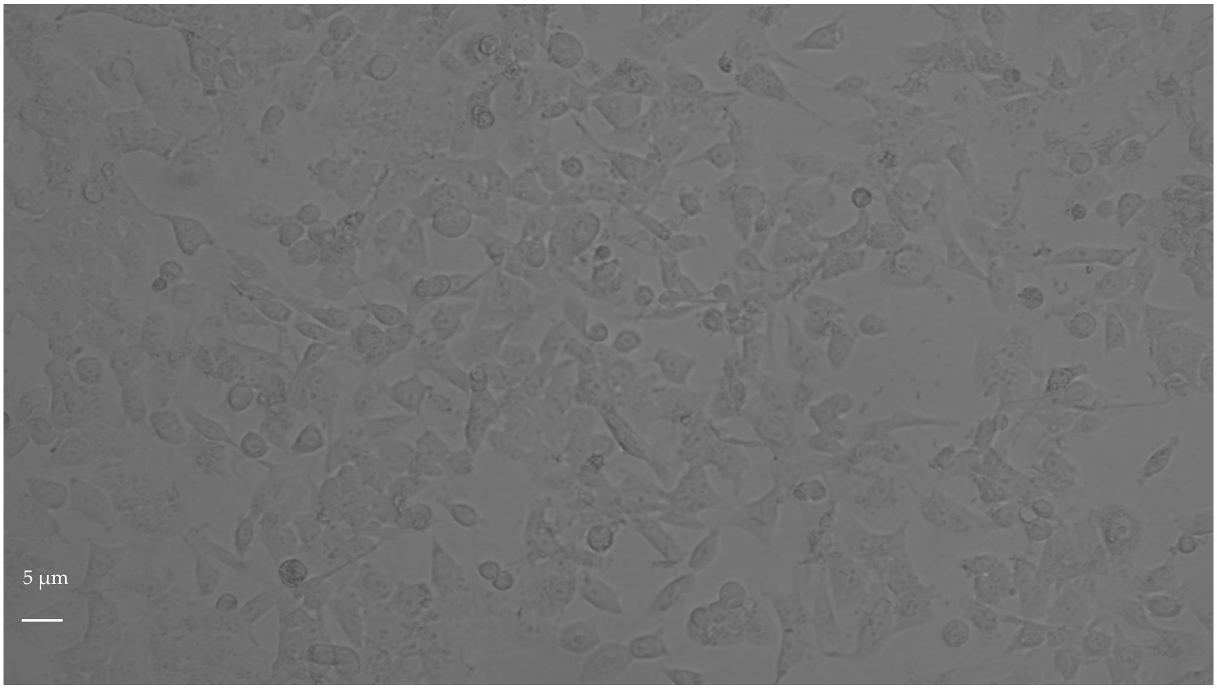

(f)

Figure S1. Optical microscopy images of NIH3T3 cells at 24, 48 and 36 hours of contact time with 20 μg/mL GF and AGP after the removal of the graphene samples. a) GF, 24 hours; b) GF 48 hours; c) GF 72 hours; d) AGP 24 hours; e) AGP, 48 hours; f) AGP, 72 hours.

Magnification 20×, scale bar= 5 μm.

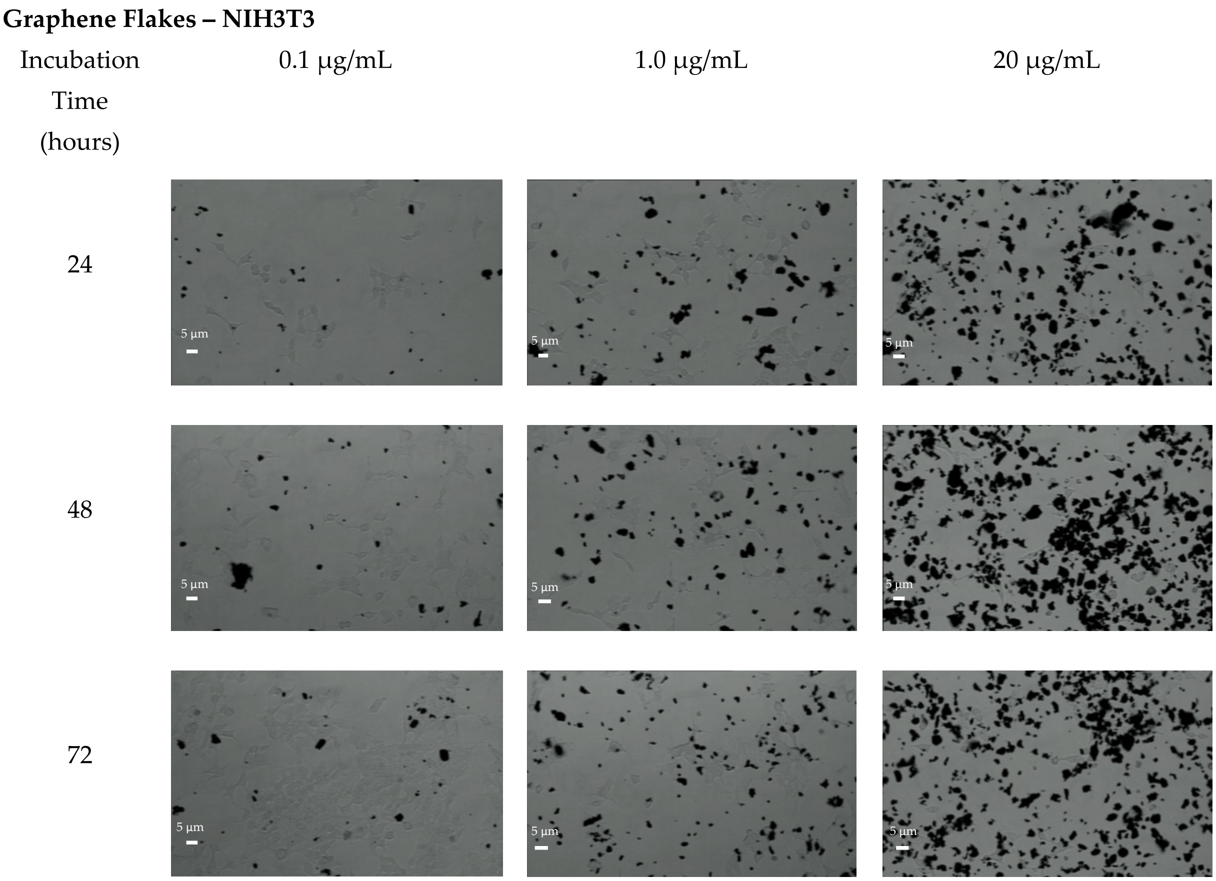
Graphene Flakes – NIH3T3
Incubation Time (hours)
0.1 μg/mL
1.0 μg/mL
20 μg/mL
24
48
72
5 μm

(a)

Aqueous Graphene Paste – NIH3T3

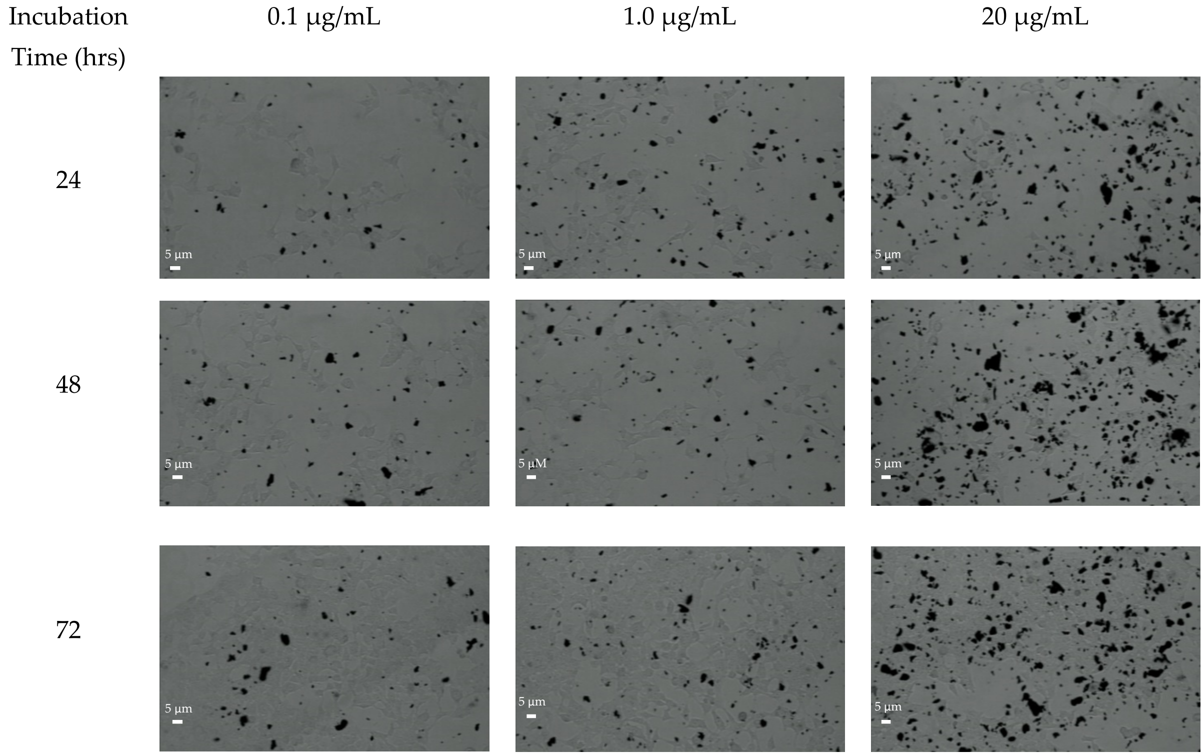

(b)

Figure S2. Optical microscopy images of NIH3T3 as a function of incubation time and concentration of Graphene Flake (a) and Aqueous Graphene Paste (b).

The density and size of aggregates increase as the concentration of GF and AGP increases. Magnification 20×. Scale bar= 5 μm.
